# Machine Learning for Dementia Prediction: A Systematic Review and Future Research Directions

**DOI:** 10.1007/s10916-023-01906-7

**Published:** 2023-02-01

**Authors:** Ashir Javeed, Ana Luiza Dallora, Johan Sanmartin Berglund, Arif Ali, Liaqat Ali, Peter Anderberg

**Affiliations:** 1 Aging Research Center, Karolinska Institutet, Tomtebodavagen, Stockholm, 17165 Solna Sweden; 2 Department of Health, Blekinge Institute of Technology, Valhallavägen 1, Karlskrona, 37141 Blekinge Sweden; 3 Department of Computer Science, University of Science and Technology Bannu, Township, Bannu, 28100 Khyber-Pakhtunkhwa Pakistan; 4 Department of Electrical Engineering, University of Science and Technology Bannu, Township, Bannu, 28100 Khyber-Pakhtunkhwa Pakistan; 5 School of Health Sciences, University of Skovde, Högskolevägen 1, Skövde, SE-541 28 Skövde Sweden

**Keywords:** Dementia prediction, Feature selection, Machine learning, Deep learning

## Abstract

Nowadays, Artificial Intelligence (AI) and machine learning (ML) have successfully provided automated solutions to numerous real-world problems. Healthcare is one of the most important research areas for ML researchers, with the aim of developing automated disease prediction systems. One of the disease detection problems that AI and ML researchers have focused on is dementia detection using ML methods. Numerous automated diagnostic systems based on ML techniques for early prediction of dementia have been proposed in the literature. Few systematic literature reviews (SLR) have been conducted for dementia prediction based on ML techniques in the past. However, these SLR focused on a single type of data modality for the detection of dementia. Hence, the purpose of this study is to conduct a comprehensive evaluation of ML-based automated diagnostic systems considering different types of data modalities such as images, clinical-features, and voice data. We collected the research articles from 2011 to 2022 using the keywords dementia, machine learning, feature selection, data modalities, and automated diagnostic systems. The selected articles were critically analyzed and discussed. It was observed that image data driven ML models yields promising results in terms of dementia prediction compared to other data modalities, i.e., clinical feature-based data and voice data. Furthermore, this SLR highlighted the limitations of the previously proposed automated methods for dementia and presented future directions to overcome these limitations.

## Introduction

Over a period of time, the advancements made in the field of medical science helped to increase the lifespan in the modern world [1]. This increased life expectancy raised the prevalence of neurocognitive disorders, affecting a significant part of the older population as well as global economies. In 2010, it was estimated that $604 billion have been spent on dementia patients in the USA alone[2]. The number of dementia patients is rapidly increasing worldwide, and statistical projections suggest that 135 million people might be affected by dementia by 2050 [3]. There are several risk factors that contribute to the development of dementia, including aging, head injury, and lifestyle. While age is the most prominent risk factor for dementia; figures suggest that a person at the age of 65 years old has 1–2% risk of developing dementia disease. By the age of 85 years old, this risk can reach to 30% [4].

**Fig. 1:**
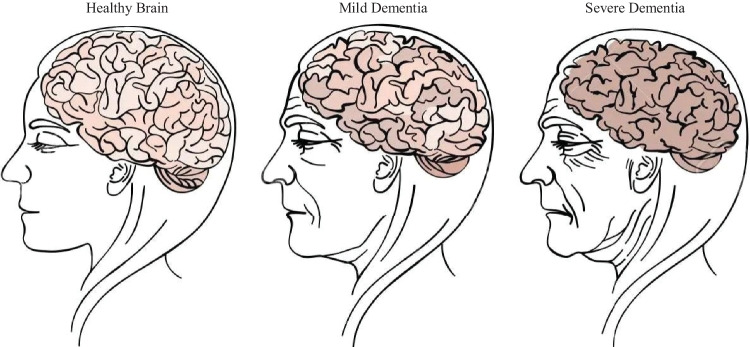
Progression of dementia disease with ageing

Dementia is a mental disorder that is characterized by a progressive deterioration of cognitive functions that can affect daily life activities such as memory, problem solving, visual perception, and the ability to focus on a particular task. Usually, older adults are most vulnerable to dementia, and people take it as an inevitable consequence of aging, which is perhaps the wrong perception. Dementia is not a part of the normal ageing process; however, it should be considered a serious form of cognitive decline that affects your daily life. Actually, the primary cause for the development of dementia is the several diseases and injuries that affect the human brain [5]. Dementia is ranked on the seventh place in the leading causes of deaths in the world [6]. Furthermore, it is the major cause of disability and dependency among older people globally [6]. A change in the person’s ordinary mental functioning and obvious signs of high cognitive deterioration are required for a diagnosis of dementia [7]. Figure 1 presents the progression of dementia with age.

### Types of dementia

**Fig. 2:**
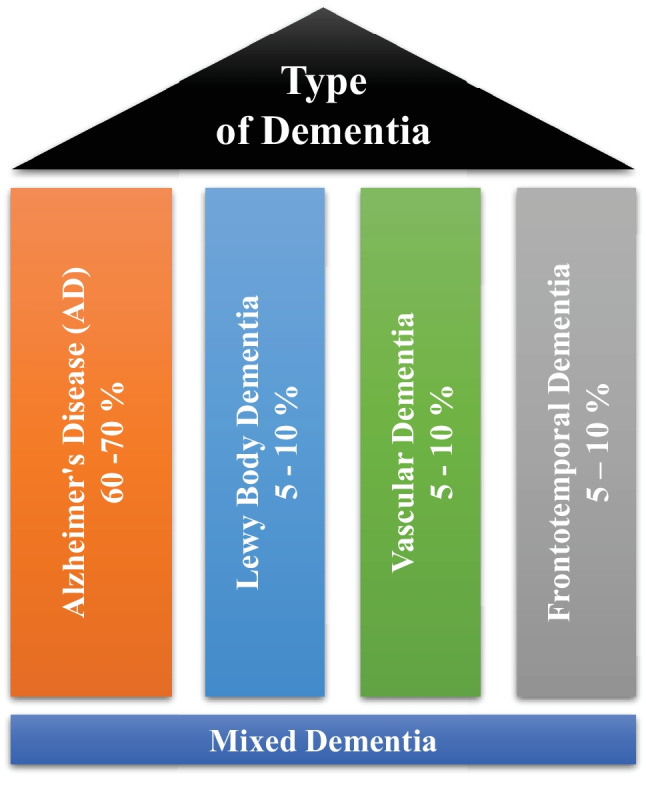
Types of dementia disease

Dementia is not a single disease, but, it is used as a generic term for several different cognitive disorders. Figure 2 provides the overview of different types of dementia along with the percentage of particular dementia type occurrence in the patients [8]. To have a better idea about dementia, we have studied common types of dementia for better problem awareness.

#### Alzheimer’s disease

Alzheimer’s disease (AD) is thought to develop when abnormal amounts of amyloid beta (A$\beta$) build up in the brain, either extracellularly as amyloid plaques, tau proteins or intracellularly as neurofibrillary tangles, affecting neuronal function, connectivity and leading to progressive brain function loss [9]. This diminished ability to eliminate proteins with ageing is regulated by brain cholesterol [10] and is linked to other neurodegenerative illnesses [11]. Except for 1–2% of cases where deterministic genetic anomalies have been discovered, the aetiology of the majority of Alzheimer’s patients remains unexplained [12]. The amyloid beta (A$\beta$) hypothesis and the cholinergic hypothesis are two competing theories presented to explain the underlying cause of AD [13].

#### Vascular dementia

Vascular dementia (VaD) is a subtype of dementia caused by problems with the brain’s blood flow, generally in the form of a series of minor strokes, which results in a slow decline of cognitive capacity [14]. The VaD refers to a disorder characterized by a complicated mix of cerebrovascular illnesses that result in structural changes in the brain, as a result of strokes and lesions, which lead to cognitive impairment. A chronological relationship between stroke and cognitive impairments is necessary to make the diagnosis [15]. Ischemic or hemorrhagic infarctions in several brain areas, such as the anterior cerebral artery region, the parietal lobes, or the cingulate gyrus, are associated with VaD. In rare cases, infarcts in the hippocampus or thalamus might cause dementia [16]. A stroke increases the risk of dementia by 70%, whereas a recent stroke increases the risk by almost 120% [17]. Brain vascular lesions can also be caused by diffuse cerebrovascular disease, such as small vessel disease [18]. Risk factors for VaD include age, hypertension, smoking, hypercholesterolemia, diabetes mellitus, cardiovascular disease, and cerebrovascular sickness; geographic origin, genetic proclivity, and past strokes are also risk factors [19]. Cerebral amyloid angiopathy, which develops when beta amyloid accumulates in the brain, can occasionally lead to vascular dementia.

#### Lewy body dementia

Lewy body dementia (LBD) is a subtype of dementia characterized by abnormal deposits of the protein alpha-synuclein in the brain. These deposits, known as Lewy bodies, affect brain chemistry, causing problems with thinking, movement, behavior, and mood. Lewy body dementia is one of the most common causes of dementia [20]. Progressive loss of mental functions, visual hallucinations, as well as changes in alertness and concentration are prevalent in persons with LBD. Other adverse effects include tight muscles, delayed movement, difficulty walking, and tremors, all of which are also signs and symptoms of Parkinson’s disease [21]. LBD might be difficult to identify. Early LBD symptoms are commonly confused with those of other brain diseases or mental problems. Lewy body dementia can occur alone or in conjunction with other brain disorders [22]. It is a progressive disorder, which means that symptoms emerge gradually and worsen with time. A timespan of five to eight years is averaged, although it can last anywhere from two to twenty years for certain people [23]. The rate at which symptoms arise varies greatly from person to person, depending on overall health, age, and the severity of symptoms.

#### Frontotemporal dementia

Frontotemporal Dementia (FTD) is a subtype of dementia characterized by nerve cell loss in the frontal and temporal lobes of the brain [24]. As a result, the lobes contract. FTD can have an impact on behavior, attitude, language, and movement. This is one of the most common dementias in people under the age of 65. FTD most commonly affects persons between the ages of 40 and 65; however, it may also afflict young adults and older individuals [25]. The lobes decrease, and behavior, attitude, language, and mobility can all be affected by FTD. FTD affects both men and women equally. Dissociation from family, extreme oniomania, obscene speech, screaming, and the inability to regulate emotions, behavior, personality, and temperament are examples of social display patterns caused by FTD [26]. The symptoms of FTD appeared several years prior to visiting a neurologist [27].

#### Mixed Dementia (MD)

Mixed dementia occurs, when more than one kind of dementia coexists in a patient, and it is estimated to happen in around 10% of all dementia cases [6]. AD and VaD dementia are the two subtypes that are most common in MD [28]. This case is usually associated with factors such as old age, high blood pressure, and brain blood vessel damage [29]. Because one dementia subtype often predominates, MD is difficult to identify. As a result, the individuals affected by MD are rarely treated and miss out on potentially life-changing medicines. MD can cause symptoms to begin earlier than the actual diagnosis of the disease and spread swiftly to affect the most areas of the brain [30].

## Method

Recently, numerous automated methods have been developed based on machine learning for early the prediction of different diseases [31–48]. This systematic literature review (SLR) presented hereby, investigates machine learning-based automated diagnostic systems that are designed and developed by scientists to predict dementia and its subtypes, such as AD, VaD, LBD, FTD and MD. We used the Preferred Reporting Items for Systematic Reviews and Meta-Analysis (PRISMA) criteria to conduct this SLR [49, 50]. A comprehensive search was conducted to retrieve the research articles that contain ML approaches to predict the development of dementia and its subtypes using three different types of data modalities (images, clinical-variables, voice).

### Aim of the study

SLRs are done to synthesize current evidence, to identify gaps in the literature, and to provide the groundwork for future studies [51]. Previous, SLRs studies have been done on automated diagnostic systems for dementia prediction based on ML approaches, which focused on a single sort of data modality. These SLR investigations did not emphasize the limits of previously published automated approaches for dementia prediction. The SLR presented herein assesses the previously proposed automated diagnostic systems based on deep learning (DL) and ML algorithms for the prediction of dementia and its common subtypes (e.g. AD, VaD, FTD, MD). The aim of this SLR is to analyse and evaluate the performance of automated diagnostic systems for dementia prediction using different data modalities. The main question is decomposed in the following sub-research questions:

1. What types of ML and DL techniques have been used by researchers to diagnose dementia?
2. Examine the methods of feature extraction or selection used by the researchers.
3. Analyze the different performance evaluation measures that are adopted by the researcher to validate the effectiveness of the proposed diagnostic system for demetnia.
4. Analyze the performance of ML models on various data types.
5. Identification of weaknesses in previously proposed ML models for dementia prediction.

**Fig. 3:**
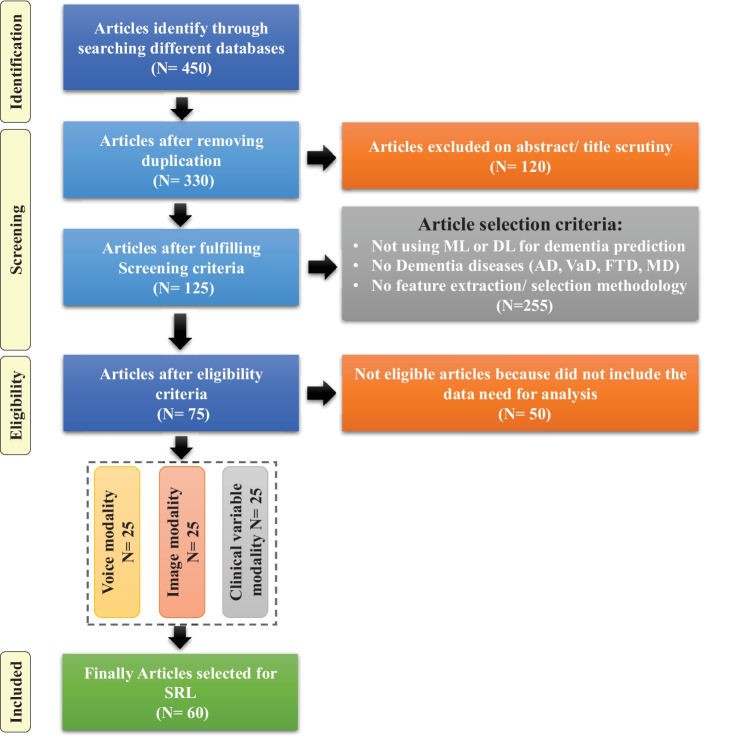
Flow diagram of PRISMA (Preferred Reporting Items for Systematic Reviews and Meta-analyses)

### Article selection

**Fig. 4:**
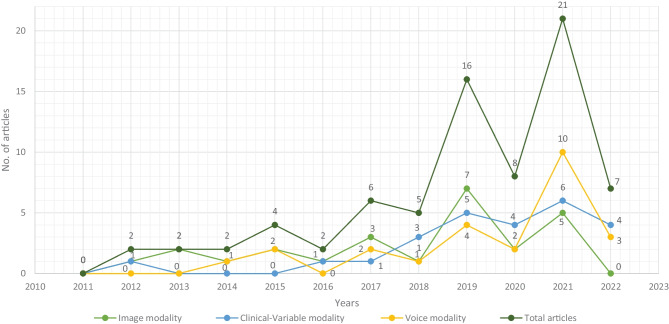
Selected research articles which are published from 2011 to 2022 regarding data modality

For this SLR study, the research articles were selected based on keywords such as ML, DL, dementia and its subtypes (AD, VaD, FTD, and MD). For the collection of research articles, we conducted an electronic search from different online databases such as ScienceDirect, PubMed, IEEE Xplore Digital Library, Springer, Hindawi, and PLOs, which helped to gather 450 research studies on the specific topic. After reviewing the title and abstract in each study, 120 publications were found to be ineligible for processing, while 330 articles were selected for further processing. Following the deduplication of data, 125 full-text publications were retrieved for further processing after the screening phase of the article selection, with 205 of them being eliminated due to not satisfying the article selection criteria of the screening phase. Finally, 50 research articles were eliminated due to not fulfilling the eligibility criteria for article selection. The final set of selected papers consisted of 75 research papers, among these final selected articles, each of the data modalities (image, clinical-variables, voice) contained 25 papers. After rerunning the database searches in May 2022, no further suitable research article was found for the selection. Figure 3 presents the workflow for article selection, which includes the four PRISMA guidelines-recommended steps such as identification, screening, eligibility, and inclusion [49, 50]. In recent years, ML scientists have shown a strong interest in designing and developing ML-based automated diagnostic systems for dementia prediction. Therefore, the number of research articles in this research area has been increased and it can be depicted from Fig. 4 where research articles are published years wise with regarding data modality. The publications utilized in this study were selected based on the following criteria:

1. Studies that present automated diagnostic systems for dementia and its common subtypes (AD,VaD, FTD, MD).
2. Studies published between 2011 and 2022.
3. Studies employing ML approaches for dementia diagnosis.
4. Studies which have utilized several data modalities.
5. Studies published in the English language.

## Machine learning for dementia

Over the years, the increasing use and availability of medical equipment has resulted in a massive collection of electronic health records (EHR) that might be utilized to identify dementia using developing technologies such as ML and DL [52]. These EHRs are one of the most widely available and used clinical datasets. They are a crucial component of contemporary healthcare delivery, providing rapid access to accurate, up-to-date, comprehensive patient information while also assisting with precise diagnosis and coordinated, efficient care [53]. Laboratory tests, vital signs, drugs, and other therapies, as well as comorbidities, can be used to identify the people at risk of dementia using the EHRs’ data [54]. In some situations, patients may also be subjected to costly and invasive treatments such as neuroimaging scans i.e., magnetic resonance imaging (MRI) and position emission tomography (PET)) and cerebrospinal fluid (CSF) collection for biomarker testing [55–57]. These tests’ findings may also be found in the EHR. According to researchers, such longitudinal clinical EHR data can be used to track the advancement of AD dementia over time [58]. Recently, several automated diagnostic systems for different diseases, such as Parkinson’s disease [59], hepatitis [47], carcinoma [41], and heart failure [60–62] prediction have been designed by employing ML and DL techniques. Inspired by this fact, the unmet demand for dementia knowledge, along with the availability of relevant huge datasets, has motivated scientists to investigate the utility of artificial intelligence (AI), which is gaining a prominent role in the area of healthcare innovation [63]. ML, a subset of AI, can model the relationship between input quantities and clinical outcomes, identify hidden patterns in enormous volumes of data, and draw conclusions or make decisions that help with more accurate clinical decision-making [51]. However, computational hypotheses generated by ML models must still be confirmed by subject matter experts in order to achieve enough precision for clinical decision-making [64].

In this SLR, we have included studies that have used ML predictive models (supervised and unsupervised) for dementia prediction and excluded studies that have used statistical methods for cohort summarization and hypothesis testing (e.g., odds ratio, chi-square distribution, Kruskal-Wallis test, and Kappa-Cohen test). Furthermore, we have referenced the data modality-based study [65] for this literature review, where we have categorized the three data modality types such as image, clinical-variable and voice. Thus, we have studied each modality-based automated diagnostic system for dementia prediction that has been proposed in the past using ML and DL.

**Table 1:** Summary of image-modality based datasets

Dataset_ID	Dataset	Samples	Variables	Dementia_Type
01	NA	373	5	AD
02	ADNI	300	4	AD
03	ALZ201	72	4	AD
04	ADNI	138	6	AD
05	NINDS-AIREN	93	62	VaD, AD
06	OASIS	373	8	AD
07	OASIS-1	216	10	AD
08	OASIS-2	373	12	AD
09	ADNI	273	8	AD
10	OASIS	420	5	AD
11	ADNI	750	10	AD
12	NA	373	15	AD
13	ADNI	506	7	AD
14	OASIS	436	NA	Dementia
15	ADNI	750	10	AD
16	OASIS	373	8	AD

### Datasets

This section explains the datasets that were used in the selected research papers for experiments and performance evaluation of the proposed automated diagnostic systems designed by the researchers using ML algorithms for dementia and its subtypes. A total of 61 datasets were studied from the selected research articles. These datasets are compiled from a wide range of organizations and hospitals throughout the world. Only a few datasets are openly available to the public, while others are compiled by researchers from various hospitals and healthcare institutes. We have only included datasets that have been used to diagnose AD, VaD, FTD, MD, and LBD using ML and DL techniques. On the basis of data modality, we have categorised the dataset into three types: images, clinical_variables and voice datasets. The datasets differ in terms of the number of variables (features) and samples. As a result, we examined each modality of the dataset one by one.

#### Image modality based datasets

There are several image datasets based on brain imaging, such as magnetic resonance imaging (MRI), collected by the researchers for the diagnosis of dementia. From the Table 1, it can be depicted that Open Access Series of Imaging Studies (OASIS) and Alzheimer’s Disease Neuroimaging Initiative (ADNI) datasets are mostly used by the researchers for the experimental purpose. OASIS aims to make neuroimaging datasets available to the scientific community for free. By gathering and openly disseminating this multimodal dataset produced by the Knight ADRC and its related researchers, they had used different samples and variables of the datasets in their research work. ADNI researchers acquire, validate, and use data such as MRI, PET imaging, genetics, cognitive assessments, CSF, and blood biomarkers as disease predictors. The ADNI website contains research information and data from the North American ADNI project, which includes Alzheimer’s disease patients, people with mild cognitive impairment, and older controls. Table 1 provides us with the following information: dataset_id, dataset name, number of samples in the particular dataset, variables in the dataset, and finally, the type of dementia.

**Table 2:** Summary of clinical-variable modality based datasets

Dataset_ID	Dataset	Samples	Variables	Dementia_Type
17	HAICDDS	1354	45	MD
18	Adnimerge	1851	49	MD
19	EMIF-AD cohort	500	4	AD
20	OASIS	373	10	Dementia
21	ADRD	44945	50	Dementia
22	multi-sensor	17	13	Dementia
23	Bremen-Ost	158	7	MCI, AD
24	microRNA	39	25	AD
25	Raman spectral	37	360	AD
26	Cheng Kung	81	10	AD
27	NA	169	14	Dementia
28	LASI-DAD	2528	30	Dementia
29	NACC	15307	258	Dementia
30	NCT	138	15	Dementia
31	Lebanon	1002	20	Dementia
32	FDG-PET	329	32	AD
33	OASIS	450	NA	AD
34	LOHAS	123	40	Dementia
35	GIST	60	6	MCI
36	ADRD	1038	35	Dementia
37	HRS	9979	52	Dementia
38	MCSA	3265	36	MD
39	MNCD	84	38	AD,FTD

#### Clinical-variables modality based datasets

Throughout the course of time, the growing usage and availability of medical devices have resulted in an overwhelming collection of clinical EHR data. Furthermore, the patient’s medical history consists of medical tests and clinical records that can be used for the prediction of diseases. Thus, the importance of clinical data emerges as a vital tool for proactive management of disease. The dataset based on clinical variables for dementia consists of medical tests that are used by doctors to check the dementia status in patients, such as the Mini Mental Status Exam (MMSE), the Montreal Cognitive Assessment (MoCA), the Telephone Interview for Cognitive Status (TICS), and the Brief Interview for Mental Status (BIMS). Clinical-variables based datasets consist of information about these medical tests along with patient personal information, i.e., age, sex, and marital status. Hereby, Table 2 provides the information regarding clinical-variables modality-based datasets that are used by the researchers for the design and development of automated diagnostic systems for dementia patients based on ML. Table 2 presents the dataset_id, dataset name, number of samples in the particular dataset, variables in the dataset, and finally the type of dementia.

#### Voice modality based datasets

Speech analysis is a useful technique for clinical linguists in detecting various types of neurodegenerative disorders affecting the language processing areas. Individuals suffering from Parkinson’s disease (PD, deterioration of voice quality, unstable pitch), Alzheimer’s disease (AD, monotonous pitch), and the non-fluent form of Primary Progressive Aphasia (PPA-NF, hesitant, non-fluent speech) may experience difficulties with prosody, fluency, and voice quality. Besides imaging and clinical-variables data, the researchers employed voice recording data to identify dementia using ML and DL algorithms. The data collection process for voice data varies from dataset to dataset, for example, in a few datasets, patients were requested to answer a prepared set of questions (interview) in a specific time interval. In a few datasets, selected neuropsychological tests were carried out, the description of each neuropsychological test was played and was followed by an answering window. Table 3 presents the dataset_id, dataset name, number of samples in the particular dataset, variables in the dataset, and finally the subtype of dementia.

**Table 3:** Summary of voice modality based datasets

Dataset_ID	Dataset	Samples	Variables	Dementia_Type
40	ADReSS	156	NA	AD
41	Aishell	120	NA	AD
42	TICS-J	123	36	Dementia
43	NA	16	192	Dementia
44	VBSD	36	254	AD
45	PAR	48	20	AD
46	ADReSS	156	NA	AD
47	NBD	09	03	Dementia
48	FHS	81	27	Dementia
49	Cheng Kung	98	8	AD
50	Aphasia	72	232	Dementia
51	EHC-Istanbul	51	30	AD
52	ADReSS	156	NA	AD
53	DementiaBank’s	1273	108	AD
54	NA	09	03	Dementia
55	Carolina Collection	21	3	AD
56	FHS	5449	NA	Dementia
57	PROMPT	120	82	Dementia
58	ADReSS	144	11	AD
59	DementiaBank’s	497	108	AD
60	AZTIAHO	70	NA	AD
61	Pitt Corpus	550	17	AD

#### Data sharing challenges

In this digital era, public health decision-making has grown progressively complicated, and the utilization of data has become critical [66]. Data are employed at the local level to observe public health and target interventions; at the national scale for resource allocation, prioritization, and planning; and at the global scale for disease burden estimates, progress in health and development measurement, and the containment of evolving global health threats [67, 68]. Van Panhuis et al. have adequately described the challenges to exchanging health data [69]. Based on our initial analysis, we built on this taxonomy to identify the hurdles related to data sharing in global public health, and we have highlighted how they may apply to each typology as given below.

1. Lack of complete data, lost data, restrictive as well as conflicting data formats, a lack of metadata and standards, a lack of interoperability of datasets (e.g., structure or “language”), and a lack of appropriate analytic solutions are examples of technical barriers encountered by health information management systems.
2. Individuals and organizations face motivational challenges when it comes to sharing data. These impediments include a lack of incentives, opportunity costs, apprehension about criticism, and disagreements over data usage and access.
3. The potential and present costs of sharing data are both economic hurdles.
4. Political obstacles are those that are built into the norms of local health governance and often emerge as regulations and guidelines. They can also entail trust and ownership difficulties.
5. Legal issues that arise as a result of data collection, analysis, and usage include questions regarding who owns or controls the data, transparency, informed permission, security, privacy, copyright, human rights, damage, and stigma.
6. Ethical constraints include a lack of perceived reciprocity (i.e., the other side will not disclose data) and proportionality (i.e., deciding not to share data based on an assessment of the risks and benefits). An overall concern is that frameworks, rules, and regulations have not kept up with technological changes that are transforming how data is collected, analyzed, shared, and used.

### ML based diagnostic models for dementia: Image modality

**Fig. 5:**
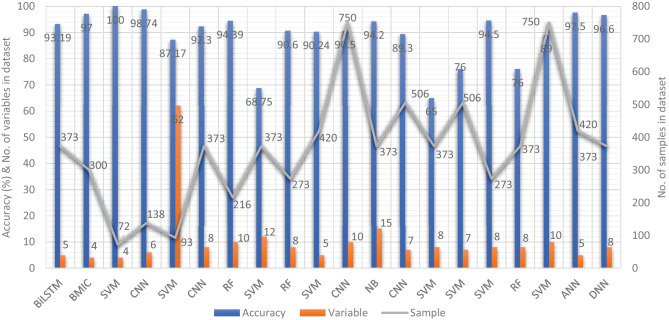
Accuracy comparison of different ML models based on image modality

In recent years, researchers have designed many ML and DL algorithms for the detection of dementia and its subtypes using MRI images of the brain. For example, Dashtipour et al. [70] proposed a ML based method for the prediction of Alzheimer’s disease. In their proposed model, they used DL techniques to extract the features from brain images, and for classification purposes, they deployed SVM and bidirectional long short-term memory (BiLSTM). Through their proposed model, they had reported the classification accuracy of 91.28%. Moreover, for early detection of the AD, a DL based approach was proposed by Helaly et al. In their proposed work, they employed convolutional neural networks (CNN). The Alzheimer’s disease spectrum is divided into four phases. Furthermore, different binary medical image classifications were used for each two-pair class of Alzheimer’s disease stages. Two approaches were used to categorize medical images and diagnose Alzheimer’s disease. The first technique employs basic CNN architectures based on 2D and 3D convolution to cope with 2D and 3D structural brain images from the ADNI dataset. They had achieved highly promising accuracies for 2D and 3D multi-class AD stage classification of 93.61% and 95.17%, respectively. The VGG19 pre-trained model had been fine-tuned and obtained an accuracy of 97% for multi-class AD stage classification [71]. Vandenberghe et al. had proposed a method for binary classification of 18F-flutemetramol PET using ML techniques for AD and mild cognitive impairment (MCI). They had tested whether support vector machines (SVM), a supervised ML technique, can duplicate the assignments made by blindfolded visual readers, as well as which image components had the highest diagnostic value according to SVM and how 18F-fluoromethylamol-based SVM classification compares to structural MRI-based SVM classification in the same cases. Their F-flutemetamol based classifier was able to replicate the assignments obtained by visual read with 100% accuracy [72]. Odusami et al. proposed a novel method for the detection of early-stage dementia from functional brain changes in MRI using a fine-tuned ResNet-18 network. Their research work presents a DL based technique for predicting MCI, early MCI, late MCI, and Alzheimer’s disease (AD). The ADNI fMRI dataset was used for analysis and consisted of 138 participants. On EMCI vs. AD, LMCI vs. AD, and MCI vs. AD, the fine-tuned ResNet18 network obtained classification accuracy of 99.99%, 99.95%, and 99.95%, respectively [73]. Zheng et al. had presented a ML based framework for differential diagnosis between VaD and AD using structural MRI features. The least absolute shrinkage and selection operator (LASSO) was then used to build a feature set that was fed into SVM for classification. To ensure unbiased evaluation of model performance, a comparative analysis of classification models was conducted using different ML algorithms to discover which one had better performance in the differential diagnosis between VaD and AD. The diagnostic performance of the classification models was evaluated using quantitative parameters derived from the receiver operating characteristic curve (ROC). The experimental finding had shown that the SVM with RBF performed well for the differential diagnosis of VaD and AD, with sensitivity (SEN), specificity (SPE), and accuracy (ACC) values of 82.65%, 87.17%, and 84.35%, respectively (AUC = 86.10–95%, CI = 0.820–0.902) [74]. Basheer et al. [75] had presented an innovative technique by making improvements in capsule network design for the best prediction outcomes. The study used the OASIS dataset with dimensions (373 X 15) to categorize the labels as demented or non-demented. To make the model swifter and more accurate, several optimization functions were performed on the variables, as well as the feature selection procedure. The claims were confirmed by demonstrating the correlation accuracy at various iterations and layers with an allowable accuracy of 92.39%. L. K. Leong and A. A. Abdullah had proposed a method for the prediction of AD based on ML techniques with the Boruta algorithm as a feature selection method. According to the Boruta algorithm, Random Forest Grid Search Cross Validation (RF GSCV) outperformed other 12 ML models, including conventional and fine-tuned models, with 94.39% accuracy, 88.24% sensitivity, 100.00% specificity, and 94.44% AUC even for the small OASIS-2 longitudinal MRI dataset [76]. Battineni et al. had presented a SVM based ML model for the prediction of dementia. Their proposed model had achieved an accuracy and precision of 68.75% and 64.18% using the OASIS-2 dataset [77]. Mathotaarachchi et al. had analyzed the amyloid imaging using ML approaches for the detection of dementia. To overcome the inherent unfavorable and imbalance proportions between persons with stable and progressing moderate cognitive impairment in a short observation period. The innovative method had achieved 84.00% accuracy and an AUC of 91.00% for the ROC [78]. Aruna and Chitra had presented a ML approach for the identification of dementia from MRI images, where they had deployed Independent Component Analysis (ICA) to extract the features from the images, and for classification purposes, SVM with different kernels is used. Through their proposed method, they had obtained an accuracy of 90.24% [79] (Fig. 5).

Supervised ML techniques and CNNs were examined by Herzog and Magoulas. They had achieved the accuracy of 92.5% and 75.0% for NC vs EMCI, 93.0% and 90.5% for NC vs. AD, respectively [80]. Battineni et al. had comprehensive applied ML model on MRI to predict Alzheimer’s disease (AD) in older subjects, and they had proposed two ML models for AD detection. In the first trial, manual feature selection was utilized for model training, and ANN produced the highest AUC of 81.20% by ROC. The NB had earned the greatest AUC of 94.20% by ROC in the second trial, which included wrapping approaches for the automated feature selection procedure [81]. Ma et al. had conducted a study where they compared feature-engineered and non-feature-engineered ML methods for blinded clinical evaluation for dementia of Alzheimer’s type classification using FDG-PET. The highest accuracy of 84.20% was obtained through CNN’s [82]. Bidani et al. had presented a novel approach in the field of DL that combines both the deep convolutional neural network (DCNN) model and the transfer learning model to detect and classify dementia. When the features were retrieved, the dementia detection and classification strategy from brain MRI images using the DCNN model provided an improved classification accuracy of 81.94%. The transfer learning model, on the other hand, had achieved an accuracy of 68.13% [83].

Moscoso et al. had designed a predictive model for the prediction of Alzheimer’s disease using MRI images. Their proposed model had obtained the highest accuracy of 84.00% [84]. Khan and Zubair had presented an improved multi-modal based ML approach for the prognosis of AD. Their proposed model had a five-stage ML pipeline, where each stage was further categorized into different sub-levels. Their proposed model had reported the highest accuracy of 86.84% using RF [85]. Mohammed et al. had evaluated the two CNN models (AlexNet and ResNet-50) and hybrid DL/ML approaches (AlexNet+SVM and ResNet-50+SVM) for AD diagnosis using the OASIS dataset. They had found that RF algorithm had attained an overall accuracy of 94%, as well as precision, recall, and F1 scores of 93%, 98%, and 96%, respectively [86]. Salvatore et al. had developed a ML method for early AD diagnosis using magnetic resonance imaging indicators. In their proposed ML model, they used PCA for extracting features from the images and SVM for the classification of dementia. They had achieved a classification accuracy of 76% using a 20-fold cross validation scheme [87]. Katako et al. had identified the AD related FDGPET pattern that is also found in LBD and Parkinson’s disease dementia using ML approaches. They studied different ML algorithms, but SVM with an iterative single data algorithm produced the best performance, i.e., sensitivity 84.00%, specificity 95.00% through 10-fold cross-validation [88]. Gray et al. had presented a system in which RF proximities were utilized to learn a low-dimensional manifold from labelled training data and then infer the clinical labels of test data that translated to this space. Their proposed model, voxel-based (FDG-PET), obtained an accuracy of 87.9% using ten-fold cross-validation [89]. Table 4 provides the overall performance evaluation of the ML models that were presented by the researchers for the prediction of dementia and its subtypes by using image data as a modality.

**Table 4:** Performance evaluation of image-modality based ML models for dementia

S.No	Authors	Year	D_ID$^{*}$	Feature	Model	Metrics	Acc.%	Spec.%	Sens.%
1	Dashtipour et al. [70]	2021	01	CNN	BiLSTM	k-fold	93.19	93.00	93.00
2	Helaly et al. [71]	2021	02	CNN	BMIC	MCC	97.00	98.00	94.00
3	Vandenberghe et al. [72]	2012	03	Voxels	SVM	MCC	100	92.00	85.00
4	Odusami et al. [73]	2021	04	ReLU	CNN	MCC	98.74	100	97.24
5	Zheng et al. [74]	2019	05	LASSO	SVM	ROC	87.17	87.17	82.65
6	Basheer et al. [75]	2019	06	KPCA	CNN	MCC	92.30	NA	NA
7	Leong et al. [76]	2019	07	Boruta	RF	MCC	94.39	100	84.24
8	Battineni et al. [77]	2019	08	RBF	SVM	MCC	68.75	NA	NA
9	Mathotaarachchi et al. [78]	2017	9	$\chi ^{2}$	RUSRF	F1-score	90.60	86.50	70.80
10	Aruna and Chitra [79]	2015	10	ICA	SVM	MCC	90.24	NA	NA
11	Herzog et al. [80]	2021	11	std, RMS	CNN	MCC	90.50	90.00	93.00
12	Battineni et al. [81]	2020	12	ANN	NB	ROC	94.20	89.56	89.92
13	Ma et al. [82]	2021	13	FPDS	CNN	MCC	89.30	67.00	84.50
14	Bidani et al. [83]	2019	14	K-means	DNN	MCC	81.94	NA	NA
15	Moscoso et al. [84]	2019	15	NA	ML	AUC	84.00	71.00	78.00
16	De Bruijne [90]	2016	16	DNN	SVM	ROC	65.00	88.00	80.00
17	Mohammed et al. [86]	2020	16	IFS	ML	ROC	90.00	93.00	98.00
18	Salvatore et al. [87]	2015	13	PCA	SVM	k-fold	76.00	NA	NA
19	Katako et al. [88]	2018	09	ISDA	SVM	AUC	94.50	95.00	84.00
20	Moscoso et al. [89]	2019	16	FSL FAST	RF	MCC	76.00	89.80	88.90
21	Tong et al. [91]	2014	11	mi-Graph	SVM	k-Fold	89.00	92.60	84.90
22	Akhila et al. [92]	2017	10	SFTA	ANN	MCC	97.50	97.50	97.50
23	Chen and Pham [93]	2013	16	mi-Graph	Markov	k-Fold	78.30	76.00	79.00
24	Patil and Yardi [94]	2013	NA	DCT	ANN	MCC	100	NA	NA
25	Gulhare et al. [95]	2017	06	niblack	DNN	MCC	96.60	NA	NA

D_ID$^*$: is a reference number of Dataset ID

### ML based diagnostic models for dementia: Clinical-variable modality

Aside from image-based ML techniques for dementia prediction, several research studies have utilized clinical-variable data with ML algorithms to predict dementia and its subtypes. For instance, Chiu et al. had designed a screening instrument to detect MCI and dementia using ML techniques. They had developed a questionnaire to assist neurologists and neuropsychologists in the screening of MCI and dementia. The contribution of 45 items that matched the patient’s replies to questions was ranked using feature selection through information gain (IG). Among the 45 items, 12 were ranked the highest in feature selection. The ROC analysis showed that AUC in test group was 94.00% [96]. Stamate et al. had developed a framework for the prediction of MCI and dementia. Their proposed framework was based on the ReliefF approach paired with statistical permutation tests for feature selection, model training, tweaking, and testing using ML algorithms such as RF, SVM, Gaussian Processes, Stochastic Gradient Boosting, and eXtreme Gradient Boosting. The stability of model performances was studied using computationally expensive Monte Carlo simulations, and the results of their proposed framework were given as for dementia detection, the accuracy was 88.00%, sensitivity was 93.00%, and the specificity was 94.00%, whereas moderate cognitive impairment had a sensitivity of 86.00% and a specificity of 90% [97]. Stamate et al. developed a system for detecting dementia subtypes (AD) in blood utilizing DL and other supervised ML approaches such as RF and extreme gradient boosting. The AUC for the proposed DL method was 85% (0.80–0.89), for XGBoost it was 88% (0.86–0.89), and for RF it was 85% (0.83–0.87). In comparison, CSF measurements of amyloid, p-tau, and t-tau (together with age and gender) gave AUC values of 78%, 83%, and 87%, respectively, by using the XGBoost [98]. Bansal1 et al. had performed the comparative analysis of the different ML methods for the detection of dementia using clinical-variables. In their experiments, they exploited the performance of four ML models, such as J48, NB, RF, and multilayer perceptrons. From the results of experiments, they had concluded that j48 outperformed the rest of the ML models for the detection of dementia [99]. Nori et al. had experimented the lasso algorithm on a big dataset of patient and identify the 50 variables by ML model with an AUC of 69.30% [100]. Alam et al. [101]used signal processing on wearable sensor data streams (e.g., electrodermal activity (EDA), photoplethysmogram (PPG), and accelerometer (ACC)) and machine learning techniques to measure cognitive deficits and their relationship with functional health deterioration.

**Fig. 6:**
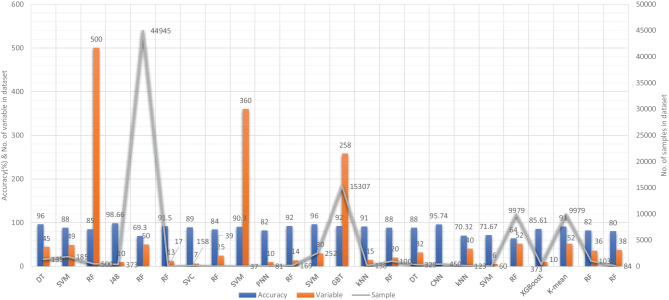
Accuracy comparison of different ML models based on clinical-variable modality

Gurevich et al. had used SVM and neuropsychological test for the classification of AD from other causes of cognitive impairment. The highest classification accuracy they had achieved through their proposed method was 89.00% [102]. Karaglani et al. had proposed a ML based automated diagnosis system for AD by using blood-based biosignatures. In their proposed method, they used mRNA-based statistically equivalent signatures for feature ranking and a RF model for classification. Their proposed automated diagnosis system had reported the accuracy of 84.60% using RF [103]. Ryzhikova et al. had analyzed cerebrospinal fluid for the diagnosis of AD by using ML algorithms. For classification purposes, artificial neural networks (ANN) and SVM discriminant analysis (SVM-DA) statistical methods were applied, with the best findings allowing for the distinguishing of AD and HC participants with 84.00% sensitivity and specificity. The proposed classification models have a high discriminative power, implying that the technique has a lot of potential for AD diagnosis [104]. Cho and Chen had designed a double layer dementia diagnosis system based on ML where fuzzy cognitive maps (FCMs) and probability neural networks (PNNs) were used to provide initial diagnoses at the base layer, and Bayesian networks (BNs) were used to provide final diagnoses at the top layer. Diagnosis results, “proposed treatment,” and “no treatment required” might be used to provide medical institutions with self-testing or secondary dementia diagnosis. The highest accuracy reported by their proposed system was 83.00% [105]. Facal et al. had studied the role of cognitive reserve in the conversion from MCI to dementia using ML. Nine ML classification algorithms were tried in their study, and seven relevant performance parameters were generated to assess the prediction accuracy for converted and non-converted individuals. The use of ML algorithms on socio-demographic, basic health, and CR proxy data allowed for the prediction of dementia conversion. The Gradient Boosting Classifier (ACC = 0.93; F1 = 0.86 and Cohen’s kappa = 0.82) and RF Classifier (ACC = 92%; F1 = 0.79 and Cohen’s kappa = 0.71) performed the best [106]. Jin et al. had proposed automatic classification of dementia from learning of clinical consensus diagnosis in India using ML techniques. All viable ML models exhibited remarkable discriminative skills (AUC >90%) as well as comparable accuracy and specificity (both around 95%). The SVM model beat other ML models by obtaining the highest sensitivity (0.81), F1 score (0.72), kappa (.70, showing strong agreement), and accuracy (second highest) (0.65). As a consequence, the SVM was chosen as the best model in their research work [107]. James et al. had evaluated the performance of ML algorithms for predicting the progression of dementia in memory clinic patients. According to their findings, ML algorithms outperformed humans in predicting incident all-cause dementia within two years. Using all 258 variables, the gradient-boosted trees approach had an overall accuracy of 92% , sensitivity of 0.45, specificity of 0.97, and an AUC of 0.92. Analysis of variable significance had indicated that just 6 variables were necessary for ML algorithms to attain an accuracy of 91% and an AUC of at least 89.00% [108]. Bougea et al. had investigated the effectiveness of logistic regression (LR), K-nearest neighbours (K-NNs), SVM, the Naive Bayes classifier, and the Ensemble Model to correctly predict PDD or DLB. The K-NN classification model exhibited an overall accuracy of 91.2% based on 15 top clinical and cognitive scores, with 96.42% sensitivity and 81% specificity in distinguishing between DLB and PDD. Based on the 15 best characteristics, the binomial logistic regression classification model had attained an accuracy of 87.5%, with 93.93% sensitivity and 87% specificity. Based on the 15 best characteristics, the SVM classification model had achieved an accuracy of 84.6% of overall instances, 90.62% sensitivity, and 78.58% specificity. A model based on NB classification obtained an accuracy of 82.05%, sensitivity of 93.10%, and a specificity of 74.41%. Finally, an ensemble model, which was constructed by combining the separate ones, attained 89.74% accuracy, 93.75% sensitivity, and 85.73% specificity [109] (Fig. 6).

Salem et al. had presented a regression-based ML model for the prediction of dementia. In their proposed method, they had investigated ML approaches for unbalanced learning. In their suggested supervised ML approach, they started by intentionally oversampling the minority class and undersampling the majority class, in order to reduce the bias of the ML model to be trained on the dataset. Furthermore, they had deployed cost-sensitive strategies to penalize the ML models when an instance was misclassified in the minority class. According to their findings, the balanced RF was the most resilient probabilistic model (with just 20 features/variables) with an F1 score of 0.82, a G-Mean of 0.88, and an AUC of 0.88 using ROC. With a F1-score of 0.74 and an AUC of 0.80 by ROC, the calibrated-weighted SVM was their top classification model for the same number of features [110]. Gutierrez et al. had designed an automated diagnosis system for the detection of AD and FTD by using feature engineering and genetic algorithms. Their proposed system had obtained the accuracy of 84% [111]. Mirzaei and Adeli had analyzed the state-of-the-art ML techniques used for the detection and classification of AD [112]. Hsiu et al. had studied ML algorithms for early identification of cognitive impairment. Their proposed model had obtained the accuracy of 70.32% by threefold cross-validation scheme [113]. Several classification models were constructed using various ML and feature selection methodologies to automate MCI detection using gait biomarkers. They had demonstrated, however, that dual-task walking differentiated between MCI and CN individuals. The ML model used for MCI pre-screening based on inertial sensor-derived gait biomarkers achieved 71.67% accuracy and 83.33% sensitivity, respectively, as reported by Shahzad et al. [114]. Hane et al. investigated the use of deidentified clinical notes acquired from multiple hospital systems over a 10-year period to enhance retrospective ML models predicting the risk of developing AD. The AUC improved from 85.00% to 94.00% by utilizing clinical notes, and the positive predictive value (PPV) rose from 45.07% (25,245/56,018) to 68.32% (14,153/20,717) in the model at the beginning of disease [115]. Table 5 provides the overall performance evaluation of the ML models that were presented by the researchers for the prediction of dementia and its subtypes by using clinical-variable data as a modality.

**Table 5:** Performance evaluation of Clinical-Variable based ML models for dementia

S.No	Authors	Year	D_ID$^{*}$	Feature	Model	Metrics	Acc.%	Spec.%	Sens.%
1	Chiu et al. [96]	2019	17	IG	DT	ROC	96.00	93.00	92.00
2	Stamate et al. [97]	2018	18	SVM	RF	k-fold	88.00	94.00	93.00
3	Visser et al. [98]	2019	19	XGBoost	RF	k-fold	85.00	NA	NA
4	Bansal et al. [99]	2018	20	WEKA	J48	k-fold	98.66	95.00	81.00
5	Nori et al. [100]	2019	21	Lasso	RF	AUC	69.30	98.70	16.40
6	Alam et al. [101]	2016	22	high-pass filter	RF	k-fold	91.50	91.80	95.70
7	Gurevich et al. [102]	2017	23	PCA	SVC	MCC	89.00	94.00	74.00
8	Karaglani et al. [103]	2020	24	mRNA	RF	ROC	84.60	NA	NA
9	Ryzhikova et al. [104]	2021	25	GA	SVM-DA	k-fold	90.30	84.00	84.00
10	Cho and Chen [105]	2012	26	FCM	PNNs	NA	82.00	NA	NA
11	Facal et al. [106]	2019	27	ANN	RF	k-fold	92.00	88.00	88.00
12	Jin et al. [107]	2021	28	NA	SVM	MCC	96.00	95.00	81.00
13	James et al. [108]	2021	29	NA	GBT	k-fold	92.00	97.00	45.00
14	Bougea et al. [109]	2021	30	NA	K-NN	AUC	91.00	85.73	93.75
15	Salem et al. [110]	2021	31	NA	RF	ROC	88.00	NA	NA
16	Garcia-Gutierrez et al. [111]	2022	32	GA	DT	F1-score	88.00	NA	NA
17	Mirzaei and Adeli [112]	2022	33	RF	CNN	k-fold	95.74	NA	NA
18	Hsiu et al. [113]	2022	34	LDA	KNN	k-fold	70.32	72.00	68.00
19	Shahzad et al. [114]	2022	35	EFA	SVM	k-fold	71.67	NA	83.33
20	Hane et al. [115]	2020	36	NA	ML	AUC	94.00	98.00	45.00
21	Aschwanden et al. [116]	2020	37	NA	RF	AUC	64.00	NA	67.00
22	Ryu et al. [117]	2020	20	HPO	XGBoost	K-fold	85.61	81.40	77.27
23	de Langavant et al. [118]	2018	37	PCA	k-mean	AUC	91.00	93.30	93.60
24	Fouladvand et al. [119]	2019	38	RNN	RF	F1-score	82.00	44.30	13.00
25	Balea-Fernandez et al. [120]	2021	39	GA	RF	AUC	80.00	71.00	100

D_ID$^*$: is a reference number of Dataset ID

### ML based diagnostic models for dementia: Voice modality

**Fig. 7:**
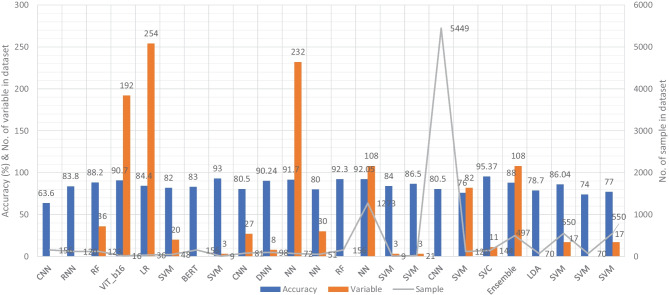
Accuracy comparison of different ML models based on voice modality

Similar to the image and clinical-variable modalities, researchers had also developed automated diagnostic systems based on voice data for the prediction of dementia. Hereby, we have reviewed the research work done by the scientists in detail. For example, Chlasta and Wolk had worked on the computer-based automated screening of dementia patients by spontaneous speech analysis using DL and ML techniques. In their work, they used neural networks to extract the features from the voice data; the extracted features were then fed into a linear SVM for classification purposes. Their SVM model had obtained the accuracy of 59.1% while CNN based ML model had reported the accuracy of 63.6% [121]. Chien et al. had presented an ML model for the assessment of AD using speech data. Their suggested model included a feature sequence that was used to extract the features from the raw audio data, as well as a recurrent neural network (RNN) for classification. Their proposed ML model had reported an accuracy of 83.80% based on the ROC curve [122]. Shimoda et al. had designed an ML model that identified the risk of dementia based on the voice feature in telephone conversations. Extreme gradient boosting (XGBoost), RF, and LR based ML models were used, with each audio file serving as one observation. The predictive performance of the constructed ML models was tested by characterizing the ROC curve and determining the AUC, sensitivity, and specificity [123]. Nishikawa et al. had developed an ensemble discriminating system based on a classifier with statistical acoustic characteristics and a neural network of transformer models, with an F1-score of 90.70% [124]. Liu et al. had introduced a new technique for recognizing Alzheimer’s disease that used spectrogram features derived from speech data, which aided families in comprehending the illness development of patients at an earlier stage, allowing them to take preventive measures. They used ML techniques to diagnose AD using speech data collected from older adults who displayed the attributes described in the speech. Their proposed method had obtained the maximum accuracy of 84.40% based on LogisticRegressionCV [125]. Searle et al. had created a ML model to assess spontaneous speech, which might potentially give an efficient diagnostic tool for earlier AD detection. Their suggested model was a fundamental Term Frequency-Inverse Document Frequency (TF-IDF) vectorizer as input into an SVM model, and the top performing models were a pre-trained transformer-based model ’DistilBERT’ when used as an embedding layer into simple linear models. The proposed model had obtained the highest accuracy of 82.00% [126]. Zhu et al. had suggested an ML model that employed the speech pause as an effective biomarker in dementia detection, with the purpose of reducing the detection, model’s confidence levels by adding perturbation to the speech pauses of the testing samples. They next investigated the impact of the perturbation in training data on the detection model using an adversarial training technique. The proposed model had achieved an accuracy of 84.00% [127]. Ossewaarde et al. had proposed ML model based on SVM for the classification of spontaneous speech of individuals with dementia based on automatic prosody analysis. Their findings suggest that the classifier can distinguish some dementia types (PPA-NF, AD), but not others (PD) [128]. Xue et al. had developed an ML model based on DL for the detection of dementia by using voice recordings. In their ML model, long short-term memory (LSTM) network and the convolutional neural network (CNN) utilized audio recordings to categorize whether the recording contained a participant with either NC or only DE and to discriminate between recordings belonging to those with DE and those without DE (i.e., NDE (NC+MCI)) [129]. Weiner et al. had presented two pipelines of feature extraction for dementia detection: the manual pipeline used manual transcriptions, while the fully automatic pipeline used transcriptions created by automatic speech recognition (ASR). The acoustic and linguistic features that they had extracted need no language specific tools other than the ASR system. Using these two different feature extraction pipelines, they had automatically detect dementia [130] (Fig. 7).

Furthermore, Sadeghian et al. had presented the empirical evidence that a combination of acoustic features from speech, linguistic features were extracted from an automatically determined transcription of the speech including punctuation, and results of a mini mental state exam (MMSE) had achieved strong discrimination between subjects with a probable AD versus matched normal controls [131]. Khodabakhsh et al. had evaluated the linguistic and prosodic characteristics in Turkish conversational language for the identification of AD. Their research suggested that prosodic characteristics outperformed linguistic features by a wide margin. Three of the prosodic features had helped to achieve a classification accuracy of more than 80%, However, their feature fusion experiments did not improve classification performance any more [132]. Edwards et al. had analyzed the text data at both the word level and phoneme level, which leads to the best-performing system in combination with audio features. Thus, the proposed system was both multi-modal (audio and text) and multi-scale (word and phoneme levels). Experiments with larger neural language models had not resulted in improvement, given the small amount of text data available [133]. Kumar et al. had identified speech features relevant in predicting AD based on ML. They had deployed neural network for the classification and obtained the accuracy of 92.05% [134]. Ossewaarde et al. had built ML model based on SVM for the classification from spontaneous speech of individuals with dementia by using automatic prosody [128]. Luz et al. had developed an ML approach for analyzing patient speech in dialogue for dementia identification. They had designed a prediction model, and the suggested strategy leveraged additive logistic regression (ML boosting method) on content-free data gathered through dialogical interaction. Their proposed model obtained the accuracy of 86.50% [135]. Sysed et al. had designed a multimodal system that identified linguistic and paralinguistic traits of dementia using an automated screening tool. Their proposed system had used bag-of-deep-feature for feature selection and ensemble model for classification [136]. Moreover, Sarawgi et al. had used multimodal inductive transfer learning for AD detection and severity. Their proposed system further achieved state-of-the-art AD classification accuracy of 88.0% when evaluated on the full benchmark DementiaBank Pitt database. Table 6 provides the overall performance evaluation of the ML models that were presented by the researchers for the prediction of dementia and its subtypes by using voice-modality data.

**Table 6:** Performance evaluation of voice-modality based ML models for dementia

S.No	Authors	Year	D_ID$^{*}$	Feature	Model	Metrics	Acc.%	Spec.%	Sens.%
1	Chlasta and Wołk [121]	2021	40	VGGish	CNN	F1-score	63.60	NA	NA
2	Chien et al. [122]	2021	41	CRNN	RNN	ROC	83.80	83.80	75.60
3	Shimoda et al. [123]	2021	42	PRAAT	RF	AUC	88.20	80.00	96.40
4	Nishikawa et al. [124]	2022	43	lightGBM	ViT_b16	F1-score	90.70	NA	87.5
5	Liu et al. [125]	2019	44	Spectrogram	LR	k-fold	84.40	91.30	87.50
6	Searle et al. [126]	2022	45	CRF	SVM	F1-score	82.00	NA	NA
7	Zhu et al. [127]	2021	46	Wav2vec	BERT	ROC	83.00	NA	NA
8	Ossewaarde et al. [128]	2019	47	VAD	SVM	ROC	93.00	NA	NA
9	Xue et al. [129]	2021	48	RNN	CNN	AUC	80.50	75.00	87.30
10	Weiner [130]	2017	49	ASR	DNN	Acc	90.24	NA	NA
11	Sadeghian et al. [131]	2017	50	VAD	NN	Acc	91.70	NA	NA
12	Khodabakhsh et al. [132]	2015	51	VAD	NN	Acc	80.00	NA	NA
13	Edwards et al. [133]	2020	52	LDA	RF	F1-score	92.30	NA	NA
14	Kumar et al. [134]	2021	53	SVC	NN	MCC	92.05	NA	NA
15	Ossewaarde et al. [128]	2019	54	VAD	SVM	ROC	84.00	NA	NA
16	Luz et al. [135]	2018	55	VGO	SVM	ROC	86.50	88.00	81.20
17	Xue et al. [129]	2021	56	LSTM	CNN	AUC	80.50	NA	NA
18	Orsulic-Jeras et al. [137]	2021	57	PCA	SVM	k-fold	76.00	NA	NA
19	Syed et al. [136]	2021	58	Fusion	SVC	Acc	95.37	94.44	96.30
20	Sarawgi et al. [138]	2020	59	PCA	Ensemble	F1-score	88.00	NA	NA
21	Calza et al. [139]	2021	NA	SSVAD v1.0	RF	F1-score	75.00	NA	NA
22	Haider et al. [140]	2019	60	eGeMAPS	LDA	F1-score	78.70	NA	NA
23	López-de-Ipiña et al. [141]	2015	61	SSF + EF	SVM	k-fold	86.04	NA	NA
24	Orimaye et al. [142]	2014	60	IG	SVM	F1-score	74.00	75.00	73.00
25	Santander-Cruz et al. [143]	2022	61	BERT	SVM	k-fold	77.00	80.00	80.00

D_ID$^*$: is a reference number of Dataset ID

## Discussion

In this SLR, we examined the research work that employed ML and DL algorithms to analyze clinical data in order to identify variables that might help predict dementia. We studied 75 research articles that were published in the last 10 years that used image, clinical-variable, and voice data to predict dementia and its subtypes. Nowadays, the healthcare industry creates a vast quantity of data on patients’ health; this data is used by researchers to enhance individual health by utilizing developing technologies such as ML and DL. As a result, researchers can not only distinguish dementia patients from healthy people with high accuracy, but also forecast the disease progression of MCI patients.

**Fig. 8:**
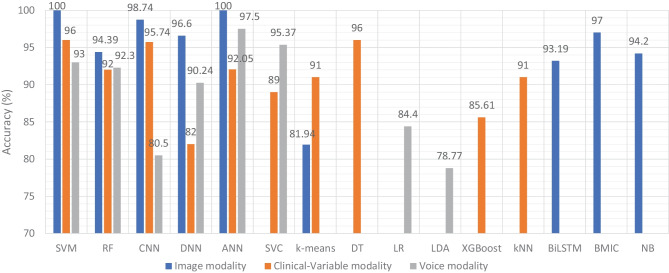
Accuracy comparison of ML models based on data modality

Therefore, researchers have expressed a strong interest in designing and developing automated diagnostic systems based on ML and DL techniques. As seen in Fig. 4., there has been an exponential increase in the number of such research publications that use ML algorithms for dementia prediction and detection in the previous four years. We investigated the selected papers using significant performance assessment criteria for ML and DL approaches such as data attributes, computational methodologies, and study emphasis. In this SLR, we have uncovered research gaps in the present literature as well as anticipated future research opportunities. Additionally, in Fig. 8 model comparison, we examined the performance of multiple ML algorithms for dementia prediction based on three types of data modalities: image, clinical-variable, and voice. The accuracy gained by image-based ML algorithms is higher when compared to clinical-variable and voice modalities, as shown in Fig. 8 model comparison. Moreover, the researchers’ suggested SVM, RF, and ANN-based ML techniques outperformed the rest of the ML algorithms in terms of performance. According to Fig. 8 model comparison, voice modality-based ML models show worse accuracy when compared to image and clinical-variable modality data. As a result, there is still a performance gap for researchers to close in order to improve the performance of ML algorithms for the prediction of dementia using voice data. Hence, researchers have shown a strong interest in the creation of automated diagnosis systems for dementia prediction utilizing speech data and ML algorithms, as illustrated in Fig. 4.

**Fig. 9:**
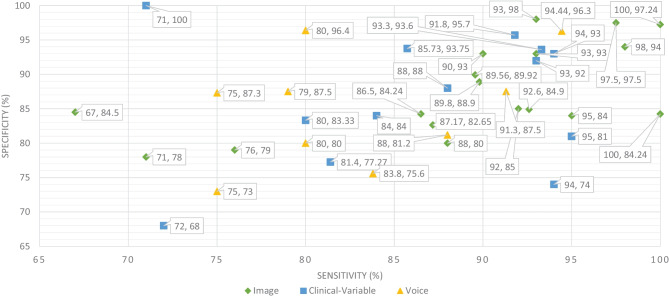
Sensitivity and specificity comparison of ML based on modality

The ML and DL models are likely prone to problems such as poor quality of data, poor selection of ML model, Bias Variance tradeoff and training too complex models. Thus, scientists have developed various evaluation metrics (i.e., ROC, AUC, MCC, F1-score, K-fold) and methods to avoid these problems. The data is a crucial element in ML because ML models work only with numeric data; therefore, poor data quality results in lower performance of ML models. Moreover, imbalance classes in the dataset also cause the bias results from the ML models. Thus, this problem can be overcome by oversampling or undersampling the training data. There are different techniques that are used by the AI engineers for oversampling, such as random oversampling and the synthetic minority oversampling technique (SMOTE). To evaluate the bias researchers’ work, use sensitivity and specificity as an evaluation metric to measure the bias of the ML model. Higher values of sensitivity and specificity means model is free from the biasness while having either one parameter value higher and other one is lower means there is biasness exist. Thus, we have also studied the sensitivity and specificity, along with the accuracy, of the previously proposed ML models for dementia prediction. Figure 9 Comparison provides a brief description of the sensitivity and specificity of the ML models for the detection of dementia based on different data modalities. From Fig. 9, we can observe that ML models have higher values for sensitivity and specificity when using image data as compared to clinical-variable and voice modality data. In comparison to accuracy from Fig. 8 to sensitivity and specificity from Fig. 9, we have noted that the results obtained from image based modality are more reliable and precise using ML and DL algorithms in spite of clinical and voice modality.

Furthermore, the correlation between sensitivity and specificity would help us understand the efficacy of the ML models, which are designed for automated disease prediction. The mathematical terms “sensitivity” and “specificity” indicate the accuracy of a test that reports the presence or absence of a disease. Individuals who meet the requirement are labelled “positive,” while those who do not are considered “negative”. The chance of a positive test, conditioned on being actually positive, is referred to as sensitivity (the true positive rate), while specificity (true negative rate) is the likelihood of a negative test if it is actually negative. Sensitivity and specificity are inversely proportional, which means that as sensitivity rises, specificity falls, and vice versa. Mathematically, sensitivity and specificity are given as:

1 $$\begin{aligned} Sensitivity = \frac{TP }{TP + FN} \end{aligned}$$

2 $$\begin{aligned} Specificity = \frac{TN }{TN+ FP} \end{aligned}$$

On the other hand, accuracy is a ratio of number of correct assessments / number of all assessments. The proportion of genuine positive outcomes (both true positive and true negative) in the selected population is represented by the numerical value of accuracy. The test result is accurate 99% of the time, whether positive or negative. For the most part, this is right. However, it is worth noting that the equation of accuracy means that even if both sensitivity and specificity are high, say 99%, this does not imply that the test’s accuracy is also high. In addition to sensitivity and specificity, accuracy is determined by the prevalence of the illness in the target population. A diagnosis for a rare ailment in the target group may have high sensitivity and specificity but low accuracy. However, for a balanced dataset, ML models with higher sensitivity and specificity result in higher accuracy. Hence, accuracy must be interpreted carefully. The mathematical formula for accuracy is given as:

3 $$\begin{aligned} Accuracy = \frac{TP + TN}{TP + TN + FP+FN} \end{aligned}$$

where, TP stands for the number of true positives, FP stands for the number of false positives, TN stands for the true negative, and FN stands for the false negative.

**Fig. 10:**
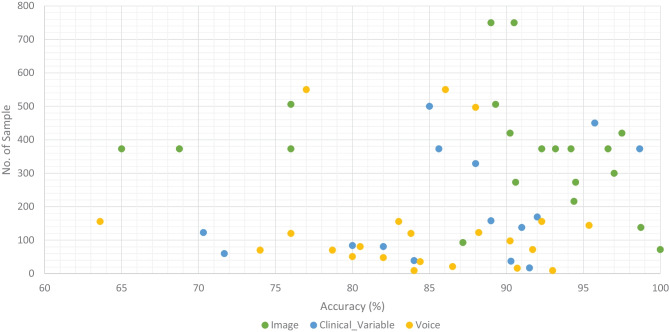
Accuracy comparison of ML models along with number of sample in the dataset based on data modality

We classified all datasets that were used by researchers to test the performance of their proposed ML models for the prediction of dementia (AD, VaD, MCI, and FTD) into three types: image, clinical-variable, and voice. A total of 61 datasets were examined in terms of the number of samples and variables in the datasets. In image modality datasets from the Table 1, it can be observed that the ADNI dataset has a significant number of samples, which is 750, while the NINDS-AIREN dataset has more variables as compared to the rest of the datasets in the image modality data. Moreover, from the Table 2 of clinical-variable modality datasets, it can be noticed that the ADRD dataset has the highest number of samples (44945) as compared to the rest of the dataset, while the Raman spectral dataset has the highest number of variables (366). In the last, Table 3 of voice data modality elaborated the dataset of voice modality where FHS dataset has highest number of samples of 5449 while VBSD dataset had highest variables of 254 as compared to rest of the datasets in voice modality. The type of data and the size of the dataset are two important factors that have a significant influence on the performance of ML models. Thus, we have also studied this factor by comparing the accuracy along with the number of samples in the dataset with respect to data modalities. From Fig. 10, it can be observed that the majority of the ML models that used image data have higher accuracy along with a higher number of samples in the dataset. There are few ML models that show poor performance when the number of samples in the dataset is large. While, clinical-variable and voice modalities show prominent performance when the number of samples in the dataset is small.

**Fig. 11:**
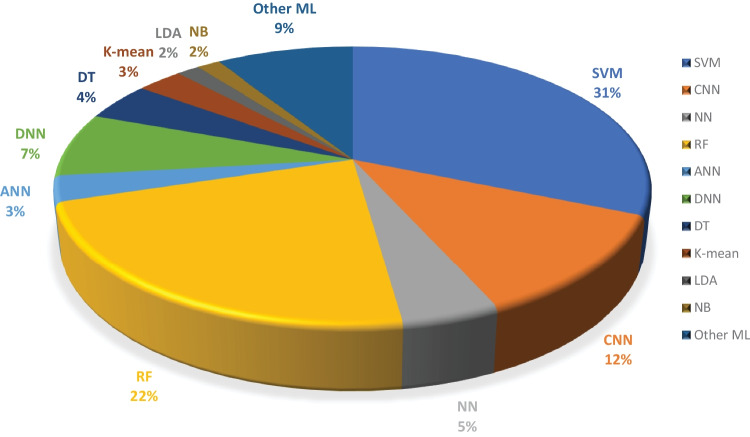
Overall percentage of ML models used in the selected research articles regardless of data modality

Moreover, we examined the effectiveness of ML classifiers utilized by the researchers in their proposed automated diagnostic systems for dementia prediction and classification. According to the selected studies of this SLR, SVM is the most commonly used ML classifier by researchers for the classification of patients and normal subjects using three data modalities (i.e., image, clinical-variable, voice), RF is the second most commonly used ML classifier by researchers, and CNN is the third most commonly used ML classifier by researchers. It can be observed from the Fig. 11. SVMs are the most powerful tools for the binary classification task, along with RF. From Fig. 8, we can see that SVM also obtained the highest average accuracy based on three types of data modalities. Hence, this factor also encourages the scientists to employ SVM as a binary classifier for dementia prediction or other disease prediction systems. From Fig. 11, we can observe the percentage of other ML classifiers that were used by the researchers in selected research articles for the automated diagnosis of dementia.

**Fig. 12:**
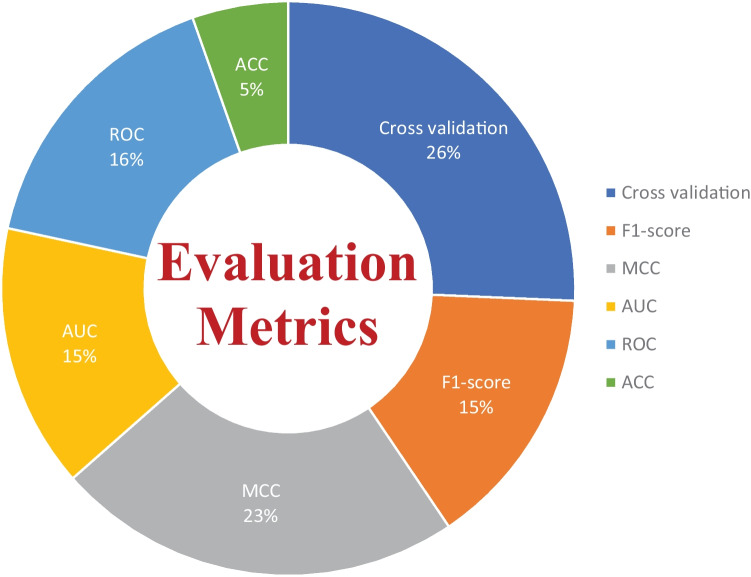
Overall percentage of evaluation metrics of ML models used by the researchers in the selected research articles

There are several evaluation metrics that are used for the performance assessment of ML models, such as F1score, AUC, ROC, Matthew’s correlation coefficient (MCC), cross-validation, K-fold, specificity, sensitivity, and accuracy. Each evaluation metric has its own pros and cons. Thus, the selection of appropriate evaluation metrics for the assessment of the ML model is essential to understanding its efficiency and performance. For instance, when data plays a vital role in ML models for decision-making and a dataset has unbalanced classes, it may be possible that results from the ML predictive model might be biassed due to the unbalanced nature of the data in the dataset. Thus, here evaluation metrics help to eliminate the factor of biasness in the results, i.e., the k-fold. The F1-score evolution metric is suitable for the classification of multiple classes in the dataset. while ROC tells us how well the ML model can differentiate binary classes. As a result, AUC and ROC reveal how effectively the probabilities from the positive classes are separated from the probabilities from the negative classes. From Fig. 12, it can be depicted that cross validation is mostly used in the studies that were selected for this SLR to evaluate the performance of proposed ML models. MCC is the second most used evaluation metric, while ROC is in third place. The proposition of other evaluation metrics used by the researchers to validate the efficiency of their proposed ML models can be observed from Fig. 12.

### Limitations in the previously proposed ML models

ML algorithms have been effectively applied to a broad range of real-world challenges, including banking, cybersecurity, transportation, and robots. They do, however, have fundamental limitations that make them inappropriate for every problem. In the clinical domain, researchers have concentrated on the supervised learning approach, developing various automated diagnostics for AD, MCI, and dementia prediction using supervised machine algorithms. From the Figs. 8 and 11, It can be noticed that supervised ML classifiers are mostly used by the researchers in the selected past research articles. Because supervised machine learning approaches have various limitations, automated diagnostic methods for dementia prediction based on supervised techniques suffer from some, if not all, of these constraints. In this part, we have examined the drawbacks of supervised ML-based techniques for dementia prediction, which are as follows:

1. The model overfitting problem affects the performance of ML models. As previously indicated, several researchers have used the k-fold cross-validation approach to evaluate the efficacy of their constructed diagnostic system. However, because of data leaks, it may result in highly biassed findings.
2. To deal with problem of imbalance classes in the dataset, Researchers and scientists had devised several techniques to eliminate the problem of imbalance classes such as random oversampling example (ROSE), synthetic minority over-sampling technique (SMOTE) and random over sampling (ROS) etc. Unfortunately, in the selected study, the researchers had not considered this factor to deal with the problem of imbalanced classes in the dataset that cause problems of bias.
3. Supervised ML models require training on a dataset; nevertheless, training on a large quantity of significant data is a hard and time-consuming job, especially for slow learning algorithms like kNN.
4. For training and testing of the ML models, researchers had used different data partitioning methods, which resulted in inconsistent comparisons of accuracy and other evaluation metrics among the proposed ML models for dementia prediction. Thus, standard data partition schemes should be adopted (holdout) for the comparison of ML models developed by the researcher for dementia prediction.
5. Another challenge with ML-based automated diagnostic systems for dementia is the time complexity of the proposed ML algorithms. The time complexity means the overall time require to complete all the computational tasks by the ML model for making a prediction. The ML model can forecast results only after it has been trained on the training data, which takes time to analyze. Furthermore, ML models include a large number of parameters that must be manually modified in the case of supervised learning. As a result, it takes a significant amount of effort and time to fine-tune the hyperparameters of the ML model in order to get higher performance.
6. DL technology has demonstrated cutting-edge performances for the prediction of various diseases in the recent years. However, DL technology needs a massive quantity of data for model training, which is a time-consuming and tough task. Due to the complexity of data models, training is quite costly. Furthermore, DL necessitates the use of pricey GPUs and hundreds of workstations, which are not effective in terms of economics.

### Future research directions

In recent years, several ML models have been presented for the prediction of AD and MCI; nevertheless, there are still certain areas that need to be explored by academics and experts. In this section, we have discussed different research areas and the future prospects of ML algorithms for dementia detection. We infer from this study that the following major parameters have a role in the efficient identification of dementia and its forms.

Data is extremely important in the case of ML-based automated detection of dementia, especially when DL models are considered. Many of the publicly available datasets, however, are modest in size. But future research should concentrate on gathering a huge number of samples for the datasets. In this SLR, we studied ML-based automated diagnostic systems for dementia prediction using three different kinds of data modalities (image, clinical_variable, voice). From Fig. 10, it can be observed that only the image modality based ML model obtained the higher accuracy along with the large size of the dataset, while the voice modality based ML model obtained the higher accuracy on a small dataset. Thus, for the researchers, there is still room available for designing and developing the automated prediction of dementia and its sub-types by using voice data. Therefore, the interest of researchers have been tremendously raised for the development of automated diagnostic systems for dementia prediction using voice data modality and this trend can be confirmed from the Fig. 4. There is still a lot room available for the improvement in design and construction of automated diagnostic systems for the dementia using clinical-variable data modality for the researchers. Because, the ML model was developed in the past using clinical-variable data, it displays mix performance by using clinical_variable modality, i.e., when the number of samples is lower in the dataset, the ML shows lower accuracy. Thus. In the future, we need to increase the number of samples in the dataset so that we have larger datasets for experimental purposes and the designed ML model can be effectively evaluated.

In selected studies of this SLR, the majority of ML algorithms belong to the supervised category of learning. While few researchers used an unsupervised ML approach for the prediction of dementia and its subtypes, Altough, unspervsied learning approaches suffer from the limitation such as less accuracy, more expensive in term of computational etc. Therefore, it will encourage scientists and researchers to design and construct new techniques and methods using supervised ML algorithms that are more precise and accurate for the prediction of dementia and its subtypes. Moreover, in this SLR, we have analysed the various ML models based on three data modalities (image, clinical-variable, and voice), and we have comprehensively compared previously proposed ML-based systems in terms of various evaluation metrics, but with different data modalities, it would be suggested that multimodal processing techniques based on ML would provide more reliable and efficient results. Hence, in the future, researchers should exploit multimodal approaches based on ML for a better prediction of dementia and its subtypes.

## Conclusion

In contrast to earlier SLR studies that examined numerous ML techniques proposed for the automated diagnosis of dementia and its subtypes (AD, VaD, FTD, and MCI) using one type of data modality, this study reviewed ML methods for dementia considering different types of data modalities such as image data, clinical variables, and voice data. The research articles published from 2011 to 2022 were gathered using different databases. It was pointed out that ML approaches based on image data modality has shown better performance compared with ML methods trained on clinical variables based data and voice data modality. Furthermore, this study critically evaluated the previously proposed methods and highlighted limitations in these methods. To overcome these limitations, this study presented future research directions in the domain of automated dementia prediction using ML approaches. We hope that this SLR will be helpful for AI and ML researchers and medical practitioners who are working in the domain of automated diagnostic systems for dementia prediction.

## Data Availability

Not applicable.
