# Correction to: Machine Learning for Dementia Prediction: A Systematic Review and Future Research Directions

**DOI:** 10.1007/s10916-024-02109-4

**Published:** 2024-09-14

**Authors:** Ashir Javeed, Ana Luiza Dallora, Johan Sanmartin Berglund, Arif Ali, Liaqat Ali, Peter Anderberg

**Affiliations:** 1 Aging Research Center, Karolinska Institutet, Tomtebodavagen, Stockholm, 17165 Solna Sweden; 2 Department of Health, Blekinge Institute of Technology, Valhallavägen 1, Karlskrona, 37141 Blekinge Sweden; 3 Department of Computer Science, University of Science and Technology Bannu, Township, Bannu, 28100 Khyber-Pakhtunkhwa Pakistan; 4 Department of Electrical Engineering, University of Science and Technology Bannu, Township, Bannu, 28100 Khyber-Pakhtunkhwa Pakistan; 5 School of Health Sciences, University of Skovde, Högskolevägen 1, Skövde, SE-541 28 Skövde Sweden

**Journal of Medical Systems (2023) 47:17**

10.1007/s10916-023-01906-7

The author name “Liaqata Ali” in the original publication of this article was incorrect. The correct name is “Liaqat Ali”

The original article has been corrected.

